# An Innovative Pubic Symphyseal Plate for the Treatment of Open-Book Injury: A Biomechanical Feasibility Study

**DOI:** 10.3390/life15111708

**Published:** 2025-11-04

**Authors:** Marx Ribeiro, Qun Zhao, Till Berk, Luis Fernando Nicolini, Eftychios Bolierakis, Klemens Horst, Johannes Greven, Philipp Kobbe, Jörg Eschweiler, Frank Hildebrand, Hatem Alabdulrahman

**Affiliations:** 1 Department for Trauma and Reconstructive Surgery, University Hospital Halle (Saale), Ernst-Grube-Straße 40, 06120 Halle (Saale), Germany; 2 Department for Trauma and Reconstructive Surgery, BG Hospital Bergmannstrost Halle (Saale), Merseburger Str. 165, 06112 Halle (Saale), Germany; 3 Department of Mechanical Engineering, Federal University of Santa Catarina, R. Lauro Linhares 1850, Florianopolis 88070-260, Brazil; 4 Department for Orthopedics, Trauma and Reconstructive Surgery, University Hospital RWTH Aachen, Pauwelsstraße 30, 52074 Aachen, Germany; 5 Department of Mechanical Engineering, Federal University of Santa Maria, Av. Roraima 1000, Santa Maria 97105-900, Brazil; 6 Department for Thorax Surgery, University Hospital RWTH Aachen, Pauwelsstraße 30, 52074 Aachen, Germany

**Keywords:** pubic symphysis, symphyseal plate, anterior pelvic ring, biomechanical stability, biomechanical test, APC, open-book injury, synthetic pelvis model, cyclic testing

## Abstract

(1) Background: This study proposes investigating the biomechanical stability of a novel 6-hole L-shaped plate for symphyseal fixation, which allows for reduction and stability in two planes. The results of the novel plate are compared to the standard plate; (2) Methods: The novel plate fixation and the standard 6-hole pubic symphyseal plate were tested with a pelvis model simulating an APC III injury. Each group of 10 pelves was subjected to a cyclic biomechanical single-leg-stance test for 30,000 cycles simulating partial bearing loading at 1 Hz, followed by a maximum load-to-failure test. The stiffness and displacement were evaluated and analyzed; (3) Results: Stiffness measurements during cyclic loading revealed no significant differences between the groups (*p* = 0.514). The cumulative plastic deformation was significantly lower in the novel plate group (*p* = 0.005). Load-to-failure testing demonstrated that both constructs exhibited similar ultimate strength, with no significant difference in the mean of maximum force between the novel (400.61 ± 44.65 N) and reference (433.02 ± 87.60 N) groups (*p* = 0.804); (4) Conclusions: The novel plate provides comparable biomechanical stability to the reference plate under the tested cyclic loading conditions, suggesting that it could be a viable alternative to the existing standard. However, further research is necessary to understand the clinical outcomes and long-term impacts.

## 1. Introduction

Open-book pelvic injuries are a significant concern in trauma, primarily due to their association with high morbidity and mortality rates that can be as high as 10.4% [1]. Complications arise from associated soft tissue injuries and hemorrhage [2]. Often, high-energy impacts generate anteroposterior compression forces leading to this kind of injury [3].

The current gold standard for managing these injuries, with disruption of the pubic symphysis and sacroiliac (SI) joint, is Open Reduction Internal Fixation (ORIF) with anterior symphyseal plate osteosynthesis [4], which has been shown to stabilize the pubic symphysis and restore pelvic integrity [5,6]. However, implant failure remains a concern, with some studies reporting failure rates of 32–80% [7,8], although this does not lead to high revision rates [8].

Dual plate fixation is a treatment option in managing open-book pelvic injuries to improve stability, especially when additional vertical stability is necessary for the posterior pelvic ring [9]. On the other hand, studies using single plates tailored for more efficient fracture reduction in anterior pelvic ring fractures show a minimization of intraoperative demands, promoting more efficient surgery and resulting in lower blood loss and quicker recovery times [10,11]. Most of these techniques are based on patient-specific 3D printing techniques [10].

However, none of these techniques addresses the problem of aligning the separated hemispheres in the horizontal and vertical position of the resulting symphysis gap. The anatomical realignment of the pelvic hemipelves is technically demanding due to the complex three-dimensional geometry and the involvement of both anterior and posterior stabilizing structures.

A novel L-shaped, self-aligning plate tested in this study was developed with a geometry intended to produce the surgical reduction by a single maneuver and to engage both the inferior and superior aspects of the symphysis area so that forces are shared between two anatomical planes.

One way to access these features is by using biomechanical tests, which are a fundamental tool for evaluating the stability of pubic symphysis plates in treating open-book pelvic injuries. These tests typically involve load-to-failure assessments and cyclic fatigue resistance, aiming to mimic the forces experienced by the pelvis during daily activities.

In addition to experimental biomechanical testing, computational mechanical characterization methods such as the Finite Element Method (FEM) and Molecular Dynamics (MD) have become important tools in biomaterials and implant research. FEM enables analysis of stress distribution (such as von Mises’ stress) and deformation at the structural level, while MD provides insights into material behavior at the molecular or nanoscale levels. Examples include FEM studies of bone fracture risk, pubic symphyseal fixation methods, and MD investigations of composite material properties [12,13,14]. These computational approaches complement laboratory testing by allowing parametric studies, visualization of local stress fields, and exploration of conditions that are difficult to reproduce experimentally.

This study aimed to evaluate a novel L-shaped plate–designed for more efficient surgery–for treating open-book injuries in comparison with a standard plate by biomechanical testing. We hypothesize that the novel plate system can provide mechanical stability comparable to the reference plate in biomechanical tests using artificial pelvis models.

## 2. Materials and Methods

### 2.1. Specimens and Instrumentation

Twenty synthetic anatomical pelvic models (Art. No. 4060, Synbone, Zizers, Switzerland) were obtained for this biomechanical study. Each of them was prepared to simulate an anteroposterior compression injury type III (APC III) according to the Young and Burgess classification. To induce this injury, the symphysis and one sacroiliac joint connection were disrupted by a standardized cut in the plastic foam of the model. The cut was made with a scalpel at the same anatomical landmark on all specimens. The depth and orientation of the transection were kept constant.

**Figure 1:**
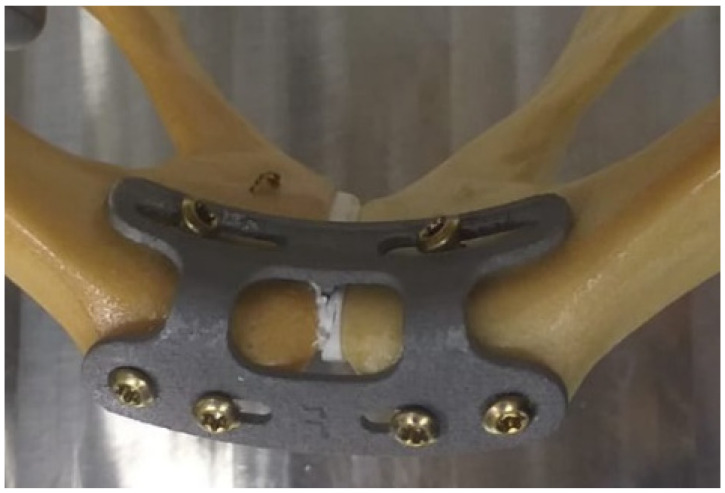
Top view of a specimen with the NSP for self-aligning pubic symphyseal osteosynthesis. The moment one side of the implant is attached, the other pelvic hemisphere can be gently pressed against the predetermined structure of the implant, thereby self-aligning both hemispheres vertically and horizontally.

The specimens were randomly assigned to one of two fixation groups: a group with a 6-hole L-shaped novel plate (Patent WO 2022/233643; US 2024/0238019), shown in Figure 1 (hereafter referred to as “Novel Symphyseal Plate (NSP)”), and a group with a 3.5 mm titanium 6-hole plate (DePuy Synthes, Zuchwil, Switzerland) (hereafter referred to as “Reference Symphyseal Plate (RSP)”).

All implantations and specimen preparations were performed by the senior attending surgeon, with care taken to ensure identical implant orientation and bicortical screw positions across specimens where possible. The implantation procedure for the RSP is a well-established surgical technique, following the open reduction and internal fixation, and is considered a gold standard for treating pubic symphysis diastasis in unstable pelvic ring injuries. Nevertheless, the NSP was positioned (as shown in Figure 1) to take advantage of the self-aligning design of the pelvic hemispheres. The procedure was to fix the plate on one hemipelvis and, in a single maneuver, the second hemipelvis itself is forced to go into anatomical reduction.

### 2.2. Biomechanical Testing

**Figure 2:**
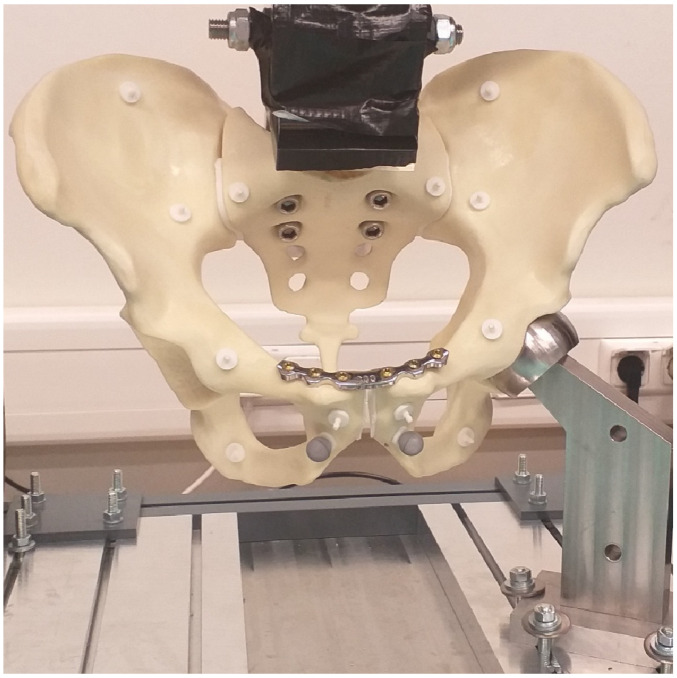
Biomechanical dynamic test setup for specimens for one-leg stance with a customized pneumatic testing machine (Dyna-Mess Prüfsysteme GmbH^®^, Stolberg, Germany). The load at the pelvis is applied to the sacrum by a custom connector and supported by the acetabulum on the same side of the simulated sacroiliac injury.

The biomechanical custom-built test setup was based on a single-leg stance configuration to replicate single-limb loading [15,16]. Each pelvis was rigidly fixed at the sacral end using a custom connector. The injured hemipelvis was supported by a custom polished semi-spherical acetabular support, allowing unconstrained rotation about its center while preventing translation, as shown in Figure 2.

Dynamic cyclic compression was applied to the sacrum via the custom connector attached to a pneumatic testing machine (Dyna-Mess Prüfsysteme GmbH^®^, Stolberg, Germany). For load-to-failure testing, an electric testing machine (Zwick-Roell GmbH & Co., KG, Ulm, Germany) with displacement control was used.

For the fatigue protocol, a sinusoidal compressive load was applied along the machine axis at the sacrum, with the pelvis oriented to reproduce a single-leg standing upright position, focusing on the stability of osteosynthetic fixation of the symphysis [16]. The compression load ranged from 5 N to 250 N at 1 Hz for 30,000 cycles. The peak load (250 N) was selected to avoid excessive plastic deformation, consistent with the load in dynamic one-leg stance studies [17,18].

Immediately following cyclic testing, specimens underwent quasi-static load-to-failure testing in displacement control at a speed of 5 mm/min. Failure was defined as a sustained drop in recorded load of >10% [19]. Setting the failure assumption at 10% of force drop is a repeatable and conservative evaluation, used as a clear signal that a component in the pelvic ring has yielded or fractured. This approach determines the ultimate strength of the bone-implant constructs.

The sample size of 10 specimens per group was defined in accordance with published biomechanical studies (see Table 1) simulating single-leg stance, where the authors used from 04 to 10 specimens per tested group [15,16,17,18,20,21,22,23,24].

### 2.3. Data Acquisition and Statistical Analysis

Axial load and actuator displacement were recorded from the testing machine controller at a sampling rate of 50 Hz. The statistical analysis was performed using open-source libraries in Python 3.12 using SciPy 1.13.1 and Statsmodels 0.14.2. These libraries complement SciPy for statistical computations, including descriptive statistics, estimation, and inference for statistical models. The normality of data distribution was assessed and proved using the Shapiro–Wilk test. Student’s *t*-test indicated significant differences between the NSP and RSP groups regarding bending stiffness, displacement, and cumulative plastic deformation due to fatigue [19]. The cumulative plastic deformation was calculated with respect to the first cycle. Moreover, bending stiffness was determined as the slope of the linear portion of the load–displacement curve.

During the dynamic test, all cycles were recorded until completion. A mixed-model ANOVA was used to compare the differences between groups (e.g., stiffness of RSP vs. NSP) and also within the group (e.g., stiffness over time) during fatigue, considering repeated measures of each cycle during the experiment. For the load-to-failure test, groups were compared using Student’s t-test. The significance level was set at 5% for all statistical tests.

## 3. Results

### 3.1. Dynamic Analysis

Stiffness, cumulative deformation, and displacement measurements were analyzed from the machine data recorded at the load point in the sacrum.

#### 3.1.1. Stiffness Analysis

**Figure 3:**
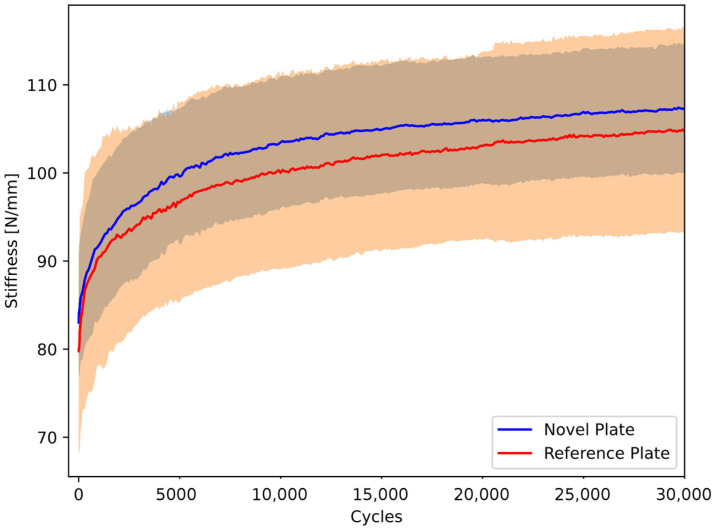
Dynamic stiffness over the cycles of artificial pelvis–implant constructs under cyclic compression, shown in terms of the mean curve (solid line) and standard deviation (shaded area) for each group separately.

**Figure 4:**
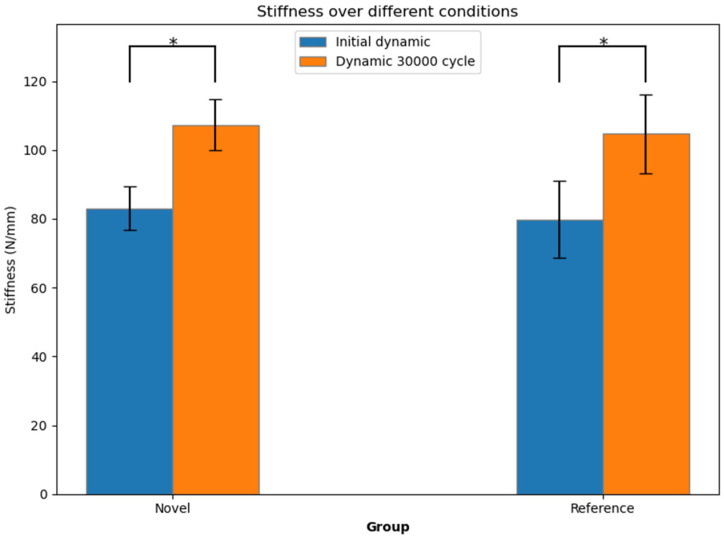
Initial dynamic stiffness and dynamic stiffness after 30,000 cycles, shown in terms of mean value and standard deviation for each group separately. Stars indicate significant differences.

The stiffness within the groups significantly increased over the cycles (*p* < 0.005), but was statistically comparable (*p* = 0.514) between the two plate groups (Figure 3 and Figure 4).

At the first testing cycle alone, the NSP mean stiffness (83.03 ± 6.29 N/mm) was compatible with that of the RSP (79.84 ± 11.30 N/mm, *p* = 0.471). After 30,000 cycles, the mean stiffness of the NSP group (107.33 ± 7.32 N/mm) remained consistent with the RSP group (104.70 ± 11.45 N/mm, *p* = 0.569).

#### 3.1.2. Cumulative Deformation Analysis

**Figure 5:**
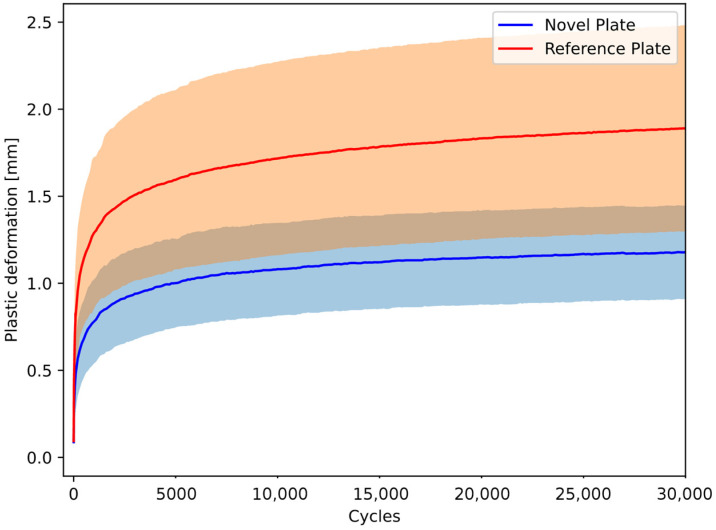
Dynamic deformation over the cycles of pelvis–implant constructs under cyclic compression is shown in terms of the mean curve (solid line) and standard deviation (shaded area) for each group separately.

The mean cumulative deformation of pelvis–implant constructs under cyclic compression gradually increased over the cycles (Figure 5). At the start of the test, both constructs had compatible cumulative deformation (0.09 ± 0.07 mm for the NSP group and 0.09 ± 0.05 mm for the RSP group, *p* = 0.826). However, after 30,000 cycles, the mean cumulative deformation of the NSP group (1.18 ± 0.27 mm) was statistically significantly lower than the RSP group (1.89 ± 0.59 mm, *p* = 0.005).

### 3.2. Load-to-Failure Analysis

**Figure 6:**
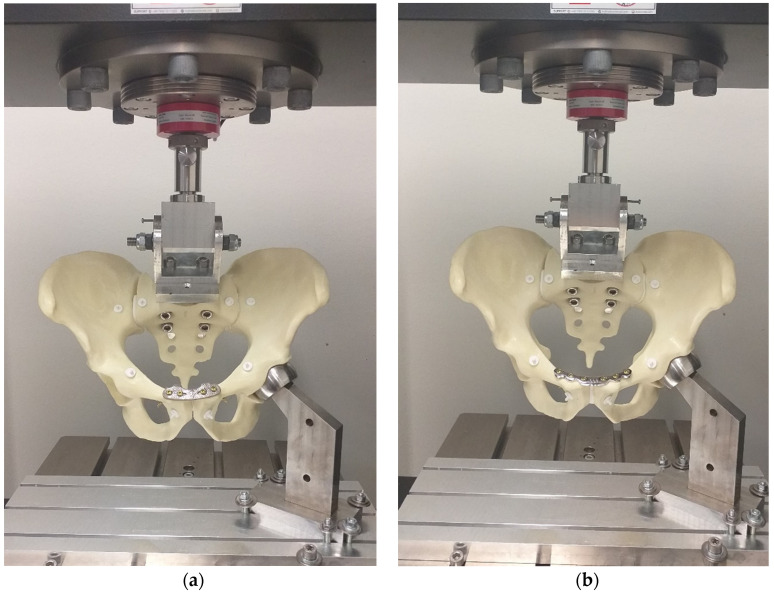
Biomechanical load-to-failure test setup for specimens with the NSP (**a**), and RSP (**b**) assembled to a material testing machine (Zwick-Roell GmbH & Co., KG, Ulm, Germany).

**Figure 7:**
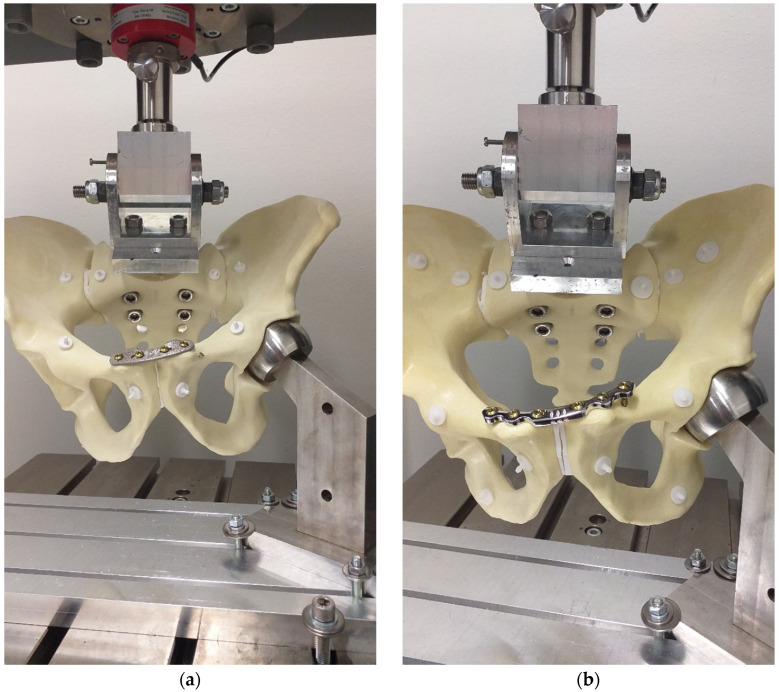
Specimens with the NSP (**a**) and the RSP (**b**) shortly before failure.

The initial positions of the load-to-failure test are presented in Figure 6, while deformed pelves shortly before failure are shown in Figure 7.

**Figure 8:**
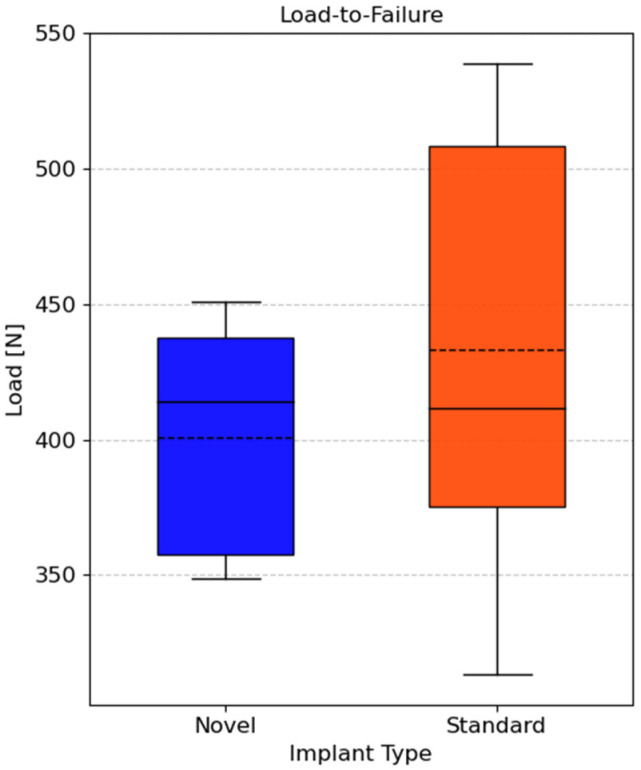
Boxplot of median maximum force of artificial pelvis–implant constructs during load-to-failure test. The mean is marked with dotted lines. No significant difference was found (*p* = 0.804).

The mean maximum force during load-to-failure testing of the NSP group (400.61 ± 44.65 N) was similar to the RSP group (433.02 ± 87.60 N, *p* = 0.804), as shown in Figure 8. The failure occurred due to deformation and loss of stability at the contralateral sacroiliac joint, rather than through implant failure. Post-test inspection revealed no screw loosening, plate deformation, or fracture in any of the tested specimens. Manual assessment confirmed joint instability on the contralateral side, indicating structural failure of the pelvic ring rather than of the fixation construct.

## 4. Discussion

This biomechanical feasibility study evaluated a 6-hole L-shaped pubic symphyseal plate compared to a 3.5 mm 6-hole plate in an artificial APC III pelvic injury. While the design aim of NSP is to facilitate intraoperative reduction, the primary objective of this study was to assess whether the NSP could provide sufficient mechanical stability for pubic symphyseal osteosynthesis. Stiffness measurements during cyclic loading revealed no statistically significant differences between the NSP and RSP groups at both the initial and final time points. These results indicate that the NSP could offer intra-operative advantages and provide comparable structural support to the RSP under the tested loading conditions, simulating partial bearing for a 3-week recovery period.

Notably, cumulative deformation—a key indicator of fatigue—was significantly lower in the NSP group compared to the RSP group. This finding suggests that the NSP has the potential to provide comparable resistance to mechanical degradation over walking cycles. Although the present study did not include a finite element model, this finding of lower plastic deformation in the NSP group is supported by the finite element study conducted by Zheng et al., where the maximum stress in a single-plate group was 127% of the yield strength of titanium alloy, which leads to plastic deformation [13]. This may be attributed to its L-shaped configuration, which enhances force distribution across the anatomical plane, thereby minimizing stress concentration points that are directly related to fatigue failure. This strategy is also evident in numerical analyses, which demonstrate that the dual-plane strategy provides superior stability and reduces mechanical stress on the plate, compared to utilizing a single plate [25].

The tendency of the NSP group towards equivalent global stability to the RSP was supported by the notion that the inferior part of the symphysis is loaded with tension [26], which the NSP provides resistance due to its dual-plane design. Varga et al. demonstrated that the utilization of the anterior double plating technique improved the stability of the inferior part of the pubic symphysis, leading to a decrease in relative movement in the SI joint when compared to a single superior plating approach [26,27].

The load-to-failure testing demonstrated that both constructs exhibited statistically comparable ultimate strength in maximum force between the NSP and RSP groups. Given that implant failure in clinical settings often occurs due to repeated cyclic loading rather than a single catastrophic overload, the improved fatigue resistance observed in the NSP may offer an advantage in long-term clinical performance.

While the L-shaped construct shows improved stability and stiffness, semi-rigid constructs have been investigated and compared with various fixation methods. This includes standard plate fixation and alternative techniques like dual plating, suture fixation, tapes, and cables [16,21,28,29,30]. For instance, Berk et al. conducted a study that demonstrated the biomechanical equivalence of suture fixation compared to plate fixation, suggesting that suture techniques could be a viable alternative [21].

Despite the promising results, this study has several limitations. First, the use of synthetic pelvis models, while standardized for biomechanical testing, does not fully replicate the properties of human bone and ligaments. Future studies should validate these findings using cadaveric models or in vivo evaluations. Additionally, the cyclic loading protocol simulated early postoperative partial weight-bearing, providing short-term insights but not long-term durability. Extended fatigue testing with higher cycle counts is needed to assess sustained implant performance. Further studies are necessary to evaluate the plate geometry for different pelvis morphologies and fracture patterns. The biological aspect also needs attention, because the risks of delayed healing or residual pain with a more constrained configuration should be addressed as potential issues.

To address these limitations and further validate our findings, future research could be conducted as a staged research program. First, cadaveric tests could reproduce physiological, multi-directional loading protocols to evaluate bone–implant interaction and long-term fatigue behavior. Second, the NSP could be compared directly with other fixation methods, while validated finite-element models are used to map implant and bone stresses and guide design improvements. Third, clinical feasibility work could assess ease of reduction, operative time, and short-term patient outcomes. If these steps confirm mechanical safety and surgical benefit, the L-shaped plate could further be evaluated for acute APC II–III injuries, as an option in revision cases after failed superior plating, or adapted for minimally invasive or patient-specific applications.

In summary, the NSP was developed aiming to facilitate anatomical reduction of pelvic hemispheres through a single maneuver during surgery. Although this surgical efficiency evaluation was out of the scope of this study, the presented biomechanical testing demonstrated that the NSP provides similar fatigue resistance and mechanical strength when compared to the RSP. Its design may lead to more efficient surgery and a reduction in the postoperative period while keeping the mechanical stability. Nevertheless, these promising results warrant further validation through cadaveric and in vivo studies to confirm long-term clinical applicability.

## 5. Conclusions

This study found that the novel 6-hole L-shaped plate presented here is as biomechanically stable as gold-standard plates, as it provides comparable mechanical stability to the tested reference plate. Beyond that, the new self-aligning system may provide features to facilitate reduction/overall operation time and prevent soft tissue damage intraoperatively. Therefore, the novel plate could be a viable alternative to the existing standard, offering adequate global stability to the pelvis with possible additional surgical efficiency. However, further research is required to understand the clinical outcomes and long-term impacts to determine the optimal indication of the novel plate in anterior pelvic fixation surgeries.

## 6. Patents

The NSP presented in this study is registered under the patents WO2022233643, US 20240238019, DE102021111653, and EP4333748.

## Figures and Tables

**Table 1:** Biomechanical studies with the type of specimen and the number of samples per group.

Author, Year	Type of Specimen	Samples per Group
Osterhoff et al., 2016 [15]	artificial	06
Jordan et al., 2021 [16]	artificial	10
Bradley et al., 2022 [17]	artificial	04
Tabaie et al., 2013 [18]	cadaveric	05
Godinsky et al., 2018 [20]	artificial	07
Berk et al., 2023 [21]	artificial	06
Goldsztajn et al., 2020 [22]	artificial	05
MacAvoy et al., 1997 [23]	cadaveric	09
Vigdorchik et al., 2012 [24]	artificial	05

## Data Availability

The data from this manuscript are available from the corresponding author upon request.

## Abbreviations

The following abbreviations are used in this manuscript:

APC	Anterior–Posterior Compression
SI	Sacroiliac
FEM	Finite Element Method
MD	Molecular Dynamics
NSP	Novel Symphyseal Plate
RSP	Reference Symphyseal Plate

## References

[1] Saleh M.H., Elashmawy A., Hazime M., Wallace B., A Saad M. (2024). Comprehensive Orthopedic Management of an Open-Book Pelvic Fracture: A Multidisciplinary Approach in Trauma Care. Cureus.

[2] Burgess A.R., Eastridge B.J., Young J.W., Ellison T.S., Ellison P.S., Poka A., Bathon G.D., Brumback R.J. (1990). Pelvic ring disruptions: Effective classification system and treatment protocols. J. Trauma..

[3] Coccolini F., Stahel P.F., Montori G., Biffl W., Horer T.M., Catena F., Kluger Y., Moore E.E., Peitzman A.B., Ivatury R. (2017). Pelvic trauma: WSES classification and guidelines. World J. Emerg. Surg..

[4] Wright R.D. (2018). Indications for Open Reduction Internal Fixation of Anterior Pelvic Ring Disruptions. J. Orthop. Trauma..

[5] Kußmaul A.C., Baur N., Wulf J., Greiner A., Neudeck R., Kistler M., Neuerburg C., Böcker W., A Becker C. (2024). Motion preservation for open book injuries of the pubic symphysis—A biomechanical cadaver study. Arch. Orthop. Trauma. Surg..

[6] Wojahn R.D., Gardner M.J. (2019). Fixation of Anterior Pelvic Ring Injuries. J. Am. Acad. Orthop. Surg..

[7] Collinge C., Archdeacon M.T., Dulaney-Cripe E., Moed B.R. (2012). Radiographic Changes of Implant Failure After Plating for Pubic Symphysis Diastasis: An Underappreciated Reality?. Clinical Orthopaedics and Related Research.

[8] Tseng K.Y., Lin K.C., Yang S.W. (2023). The radiographic outcome after plating for pubic symphysis diastasis: Does it matter clinically?. Arch. Orthop. Trauma. Surg..

[9] Pizanis A., Garcia P., Santelmann M., Culemann U., Pohlemann T. (2013). Reduction and fixation capabilities of different plate designs for pubic symphysis disruption: A biomechanical comparison. Injury.

[10] Hung C.-C., Li Y.-T., Chou Y.-C., Chen J.-E., Wu C.-C., Shen H.-C., Yeh T.-T. (2019). Conventional plate fixation method versus pre-operative virtual simulation and three-dimensional printing-assisted contoured plate fixation method in the treatment of anterior pelvic ring fracture. Int. Orthop..

[11] Wang C., Chen Y., Wang L., Wang D., Gu C., Lin X., Liu H., Chen J., Wen X., Liu Y. (2020). Three-dimensional printing of patient-specific plates for the treatment of acetabular fractures involving quadrilateral plate disruption. BMC Musculoskelet. Disord..

[12] Sass J.O., Saemann M., Kebbach M., Soodmand E., Wree A., Bader R., Kluess D. (2024). The Morphology of the Femur Influences the Fracture Risk during Stumbling and Falls on the Hip—A Computational Biomechanical Study. Life.

[13] Zheng Y.-Q., Chen L.-L., Shen J.-Z., Gao B., Huang X.-C. (2022). Biomechanical evaluation of seven fixation methods to treat pubic symphysis diastasis using finite element analysis. J. Orthop. Surg. Res..

[14] Heydari S.F., Shahgholi M., Karimipour A., Salehi M., Galehdari S.A. (2024). The effects of graphene oxide nanoparticles on the mechanical and thermal properties of polyurethane/polycaprolactone nanocomposites; a molecular dynamics approach. Results Eng..

[15] Osterhoff G., Tiziani S., Hafner C., Ferguson S.J., Simmen H.-P., Werner C.M.L. (2016). Symphyseal internal rod fixation versus standard plate fixation for open book pelvic ring injuries: A biomechanical study. Eur. J. Trauma Emerg. Surg..

[16] Jordan M.C., Jäckle V., Scheidt S., Gilbert F., Hölscher-Doht S., Ergün S., Meffert R.H., Heintel T.M. (2021). Trans-obturator cable fixation of open book pelvic injuries. Sci. Rep..

[17] Bradley H., Pierce B.B., O’NEill D., Jo C.-H., Ahn J., Farahani F.B., Greif C.B., Sanders D., Starr A. (2022). Biomechanical Comparison of 4 Transsacral Fixation Constructs in a Type 61C, Zone II Pelvic Fracture Model. J. Orthop. Trauma..

[18] Tabaie S.A., Bledsoe J.G., Moed B.R. (2013). Biomechanical Comparison of Standard Iliosacral Screw Fixation to Transsacral Locked Screw Fixation in a Type C Zone II Pelvic Fracture Model. J. Orthop. Trauma..

[19] Brandes L.L., Nicolini L.F., Greven J., Lichte P., Stopinski T.T., Sattler M., Hildebrand F., Pishnamaz M. (2021). Biomechanical Performance of BoneHelix® Compared with Elastic Stable Intramedullary Nailing (ESIN) in a Pediatric Tibia Fracture Model. Life.

[20] Godinsky R.J., Vrabec G.A., Guseila L.M., Filipkowski D.E., Elias J.J. (2018). Biomechanical comparison of locked versus non-locked symphyseal plating of unstable pelvic ring injuries. Eur. J. Trauma Emerg. Surg..

[21] Berk T., Zderic I., Varga P., Schwarzenberg P., Berk K., Grüneweller N., Pastor T., Halvachizadeh S., Richards G., Gueorguiev B. (2023). Substitutional semi-rigid osteosynthesis technique for treatment of unstable pubic symphysis injuries: A biomechanical study. Eur. J. Trauma Emerg. Surg..

[22] Goldsztajn F., Mariolani J.R.L., Belangero W.D. (2020). Placas anteriores são mais efetivas do que parafusos iliossacrais na fixação da articulação sacroilíaca? Estudo Biomecânico. Rev. Bras. Ortop..

[23] MacAvoy M.C., McClellan R.T., Goodman S.B., Chien C.-R.D., Allen W.A., van der Meulen M.C.H. (1997). Stability of Open-Book Pelvic Fractures Using a New Biomechanical Model of Single-Limb Stance. J. Orthop. Trauma..

[24] Vigdorchik J.M., O Esquivel A., Jin X., Yang K.H., A Onwudiwe N., Vaidya R. (2012). Biomechanical stability of a supra-acetabular pedicle screw Internal Fixation device (INFIX) vs External Fixation and plates for vertically unstable pelvic fractures. J. Orthop. Surg. Res..

[25] Yao F., He Y., Qian H., Zhou D., Li Q. (2015). Comparison of biomechanical characteristics and pelvic ring stability using different fixation methods to treat pubic symphysis diastasis a finite element study. Medicine.

[26] Stuby F.M., Lenz M., Doebele S., Agarwal Y., Skulev H., Ochs B.G., Zwingmann J., Gueorguiev B. (2017). Symphyseal fixation in open book injuries cannot fully compensate anterior SI joint injury—A biomechanical study in a two-leg alternating load model. PLoS ONE.

[27] Varga E., Hearn T., Powell J., Tile M. (1995). Effects of method of internal fixation of symphyseal disruptions on stability of the pelvic ring. Injury.

[28] Moreau P.E., Bokhari A., El Yahiouni S., Manach Q., Upex P., Riouallon G. (2024). Pubic symphysis tethering technique under endoscopic approach for treatment of pelvic open-book injury: A cadaver study. Trauma. Case Rep..

[29] Kußmaul A.C., Schwaabe F., Kistler M., Gennen C., Andreß S., Becker C.A., Böcker W., Greiner A. (2022). Novel minimally invasive tape suture osteosynthesis for instabilities of the pubic symphysis: A biomechanical study. Arch. Orthop. Trauma. Surg..

[30] Kußmaul A.C., Schwaabe F., Kistler M., Jörgens M., Schreyer K.F., Greiner A., Böcker W., Becker C.A. (2023). Tape suture constructs for instabilities of the pubic symphysis: Is the idea of motion preservation a suitable treatment option? A cadaver study. Arch. Orthop. Trauma. Surg..

